# Supplementary material for: A Novel Flower-Like Ag/AgCl/BiOCOOH Ternary Heterojunction Photocatalyst: Facile Construction and Its Superior Photocatalytic Performance for the Removal of Toxic Pollutants
Source: Nanomaterials (Basel). 2019 Nov 4;9(11):1562. doi: 10.3390/nano9111562 (PMC6915398; doi:10.3390/nano9111562)
Supplement: Supplementary file 1 [file nanomaterials-09-01562-s001.pdf]

A novel flower-like Ag/AgCl/BiOCOOH ternary heterojunction photocatalyst: Facile construction and its superior photocatalytic performance for the removal of toxic pollutants

Shijie Li[1,*], Bing Xue[1,2], Genying Wu[3], Yanping Liu[1,2,*] Huiqiu Zhang[1], and Deyun Ma[4]

[1] *Key Laboratory of key technical factors in Zhejiang seafood health hazards, Institute of Innovation & Application, Zhejiang Ocean University, Zhoushan, Zhejiang Province, 316022, China. lishijie@zjou.edu.cn (S. L.); xb1725621827.@163.com (B. X.); zhanghuiqiu2006@163.com (H. Z.);*

[2] *Department of Environmental Engineering, Zhejiang Ocean University, Zhoushan, Zhejiang Province, 316022, China; liuyp@zjou.edu.cn (Y. L.).*

[3] *Longquan Branch of Lishui Municipal Ecological Environment Bureau, Longquan, Zhejiang Province, 323700, China. hannah29@163.com (G. W.).*

[4] *School of Food and Pharmaceutical Engineering, Zhaoqing University, Zhaoqing, Guangdong Province, 526061, China. mady@zqu.edu.cn (D. M.).*

* *Correspondence: lishijie@zjou.edu.cn (S. L); liuyp@zjou.edu.cn (Y. L.).*

Figure

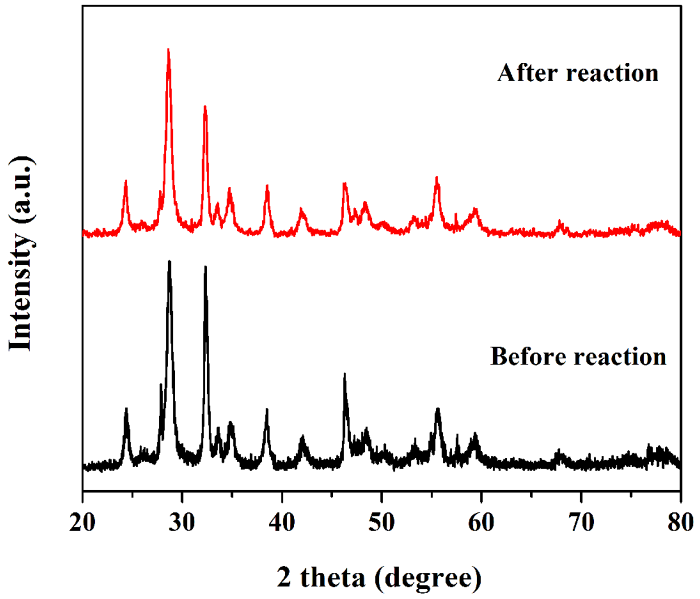

Figure S1 XRD patterns of the original and recovered Ag/AgCl/BOCH-3.
